# Ceramides and glucosylceramides are independent antagonists of insulin action

**DOI:** 10.1186/1753-6561-6-S3-P72

**Published:** 2012-06-27

**Authors:** Jose A Chavez, Siew Tein Wang, Puck Wee Chan, Scott A Summers

**Affiliations:** 1 Stedman Center for Nutrition and Metabolism Research, Duke University Medical Center, Durham, NC 27704, USA; 2 Program in Cardiovascular and Metabolic Disorders, Duke-NUS Graduate Medical School, Singapore, Singapore

## 

Ceramides and glucosylceramides (GCs) have both been implicated in the development of insulin resistance and the pathogenesis of metabolic diseases associated with obesity. Inhibitors of either ceramide or glucosylceramide production enhance insulin signaling in adipocytes and protect mice from high-fat diet induced-insulin resistance, diabetes, and hepatic steatosis. Moreover, the addition of either ceramides or glucosylceramides to 3T3-L1 adipocytes impairs insulin signaling. Since inhibitors of ceramide biosynthesis deplete cells of glucosylceramides, and since exogenous ceramides can be re-glucosylated to produce glucosylceramides, it remains a formal possibility that glucosylated ceramides, and not ceramides themselves, antagonize insulin action. In the present work, we present evidence revealing that glucosylceramides and ceramides are independent and separable antagonists of insulin signaling. The conclusion derives from the fact the two sphingolipids have differential effects on insulin signaling to Akt/PKB in cultured myotubes, but have comparable effects on insulin signaling to Akt/PKB in adipocytes. First, though inhibitors of ceramide biosynthesis prevent the antagonism of insulin signaling by saturated fatty acids in C2C12 myotubes, inhibitors of glucosylceramide synthase (GCS) have no effect. Second, incubation of C2C12 myotubes with exogenous ceramides antagonizes insulin signaling, while the addition of the ganglioside GM3 does not. And third, adenovirus-mediated overexpression of human GCS (hGCS) in myotubes protects cells from palmitate-induced inhibition of insulin signaling. In contrast, increasing glucosylated ceramide levels in 3T3-L1 adipocytes has profound effects. For example, both GM3 addition or hGCS overexpression downregulates insulin signaling in that cell type. These observations reveal that both glucosylceramides only induce insulin resistance in a subset of cell types, and confirm that both glucosylceramides and ceramides are independent antagonists of insulin action.

